# Antibiotic resistance as a global threat: Evidence from China, Kuwait and the United States

**DOI:** 10.1186/1744-8603-2-6

**Published:** 2006-04-07

**Authors:** Ruifang Zhang, Karen Eggleston, Vincent Rotimi, Richard J Zeckhauser

**Affiliations:** 1 Goldman Sachs International, Global Investment Research, London, UK; 2 Tufts University Economics Department, Medford, MA 02155, USA; 3 Department of Microbiology, Faculty of Medicine, Kuwait University, Kuwait; 4 Harvard University Kennedy School of Government, Cambridge, MA, USA

## Abstract

**Background:**

Antimicrobial resistance is an under-appreciated threat to public health in nations around the globe. With globalization booming, it is important to understand international patterns of resistance. If countries already experience similar patterns of resistance, it may be too late to worry about international spread. If large countries or groups of countries that are likely to leap ahead in their integration with the rest of the world – China being the standout case – have high and distinctive patterns of resistance, then a coordinated response could substantially help to control the spread of resistance. The literature to date provides only limited evidence on these issues.

**Methods:**

We study the recent patterns of antibiotic resistance in three geographically separated, and culturally and economically distinct countries – China, Kuwait and the United States – to gauge the range and depth of this global health threat, and its potential for growth as globalization expands. Our primary measures are the prevalence of resistance of specific bacteria to specific antibiotics. We also propose and illustrate methods for aggregating specific "bug-drug" data. We use these aggregate measures to summarize the resistance pattern for each country and to study the extent of correlation between countries' patterns of drug resistance.

**Results:**

We find that China has the highest level of antibiotic resistance, followed by Kuwait and the U.S. In a study of resistance patterns of several most common bacteria in China in 1999 and 2001, the mean prevalence of resistance among hospital-acquired infections was as high as 41% (with a range from 23% to 77%) and that among community- acquired infections was 26% (with a range from 15% to 39%). China also has the most rapid growth rate of resistance (22% average growth in a study spanning 1994 to 2000). Kuwait is second (17% average growth in a period from 1999 to 2003), and the U.S. the lowest (6% from 1999 to 2002). Patterns of resistance across the three countries are not highly correlated; the most correlated were China and Kuwait, followed by Kuwait and the U.S., and the least correlated pair was China and the U.S.

**Conclusion:**

Antimicrobial resistance is a serious and growing problem in all three countries. To date, there is not strong international convergence in the countries' resistance patterns. This finding may change with the greater international travel that will accompany globalization. Future research on the determinants of drug resistance patterns, and their international convergence or divergence, should be a priority.

## 

In 1942, the first U.S. patient with streptococcal infection was miraculously cured with a small dose of penicillin. Sixty years later, penicillin-resistant Streptococcus is widespread. Such antimicrobial resistance threatens the health of many throughout the world, since both old and new infectious diseases remain a formidable public health threat.

Among the issues that merit further scrutiny for understanding the possible spread of antimicrobial resistance, few are as salient as the impact of globalization. Clearly the movement of people and goods around the globe contributes to transmission of disease [1,2]. To what extent drug resistance and globalization are similarly related remains unclear. The breakout of Severe Acute Respiratory Syndrome (SARS) in the spring of 2003 illustrates how an infectious disease with limited therapeutic options can spread rapidly across national borders. With globalization booming, it is important to understand international patterns of resistance. If countries already experience similar patterns of resistance, it may be too late to worry about international spread. If large countries or groups of countries that are likely to leap ahead in their integration with the rest of the world – China being the standout case – have high and distinctive patterns of resistance, then a coordinated response could help substantially to control the spread of resistance. The literature to date provides only limited evidence on these issues.

We study the pattern of antibiotic resistance in specific countries to gauge the range and depth of this global health threat. China and the U.S. stand out as good choices for study. Both are world economic powerhouses increasingly responding to the forces of economic globalization. In addition, both are major consumers of antibiotics, with the U.S. also being a leading source of new antibiotics. On the other hand, it would also be interesting to compare patterns of antibiotic resistance in smaller countries that stand relatively distant from these two. Accordingly, we compare the experiences of the U.S. and China with new data on the resistance experience of Kuwait.

The first section gives brief background on antibiotic resistance and its costs. We then turn to a detailed comparison of surveillance data from China, Kuwait, and the U.S. We conclude with a plea for more research and attention on this critical issue for health and globalization.

## Background: The challenge of antimicrobial resistance

According to laws of Darwinian evolution, antimicrobial use creates a selection pressure on microorganisms: weak ones are killed, but stronger ones might adapt and survive. When pathogenic microorganisms can multiply beyond some critical mass in the face of invading antimicrobials, treatment outcome is compromised; this phenomenon is referred as antimicrobial resistance (AMR) [3-9]. This paper focuses on antibiotic resistance, a major form of AMR.

Resistance mechanisms may develop over months or years [6]. Once established, a single resistance mechanism can often allow a bacterium to resist multiple drugs. It remains unclear whether resistance is reversible, and thus whether drug effectiveness is a renewable or non-renewable resource [10-15]. Drug resistance raises the cost of treatment for infectious diseases, sometimes manifold, as well as increasing morbidity and mortality from such diseases [16-23].

The greatest long-term threat of AMR is that resistant strains erode drug efficacy over time. The development of drug-resistant *Staphylococci aureus *(SAU) well illustrates the see-saw battle between pathogens and drugs. SAU is a bacterium that harmlessly lives in the human body but can cause infections on wounds or lesions. After the clinical application of penicillin in the 1940s, SAU soon adapted to the treatment mechanism of penicillin, and by the 1950s, almost half of SAU strains had become resistant to penicillin. A new antibiotic, methicillin, was developed in the 1960s. Yet by the late 1970s, methicillin-resistant SAU, i.e. MRSA, again became widespread. Today MRSA has become a major infectious culprit that can only be effectively treated with vancomycin, one of the few last killers of superbugs. Unfortunately, in 1996, a Japanese hospital reported the first case of vancomycin-resistant SAU (VRSA) during surgery on a four-month-old boy. The U.S., France and Hong Kong subsequently all reported VRSA incidents. A few years later in 2000, linezolid was launched as a new antibiotic to combat both MRSA and VRSA. But only one year later, Boston researchers reported the first case of linezolid-resistant MRSA in an 85-year-old man undergoing peritoneal dialysis. After failing to contain his MRSA by linezolid, researchers tried five antibiotics (ampicillin, azithromycin, gentamicin, levofloxacin, and quinupristin-dalfopristin) but the unlucky man eventually died from the uncontrollable infection [24].

**Figure 1:**
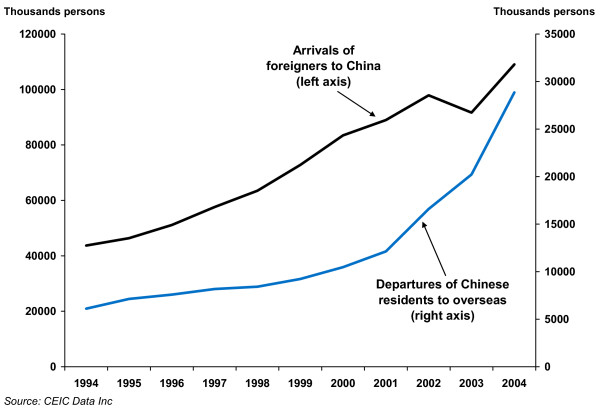
Travel to and from China has increased tremendously over the past decade.

Resistant pathogens within a hospital or specific community can spread to a nation at large or across national boundaries. Thus, for example, rapidly increasing travel and migration within China probably contributes to the growth of that nation's resistance problem. It may also spur the spread of China's resistance problems overseas as globalization greatly increases travel from and to that nation (see Figure 1).

## Methods

We collected data on drug resistance in China, the U.S. and Kuwait, drawing from published studies, reports from national surveillance systems, and previously unpublished data from a large hospital in Kuwait. Such data must be viewed with caution. Differences between countries arise not only from genuine differences in prevalence, but also from differences in sampling strategies, laboratory processing, and standards for defining a "resistant" strain. Moreover, within-country comparisons across time are biased by measurement error, particularly for small samples. However, analysis of the currently available data does yield some evidence and may help to raise awareness and efforts to improve the data and methods for addressing the problem.

Our primary measure is the prevalence of resistance by a specific bacterium to a specific drug. The prevalence is calculated as the number of resistant isolates divided by the number of total isolates collected, multiplied by 100. We compute growth rates of resistance to specific bacteria using standard year-on-year growth calculations. Where appropriate, we smooth variance in small-sample data series by using three-year running averages.

We also develop methods to aggregate specific "bug-drug" data to summarize the resistance pattern for each country. These measures weight resistance rates by (1) the isolation frequency for each bacterium (that is, the proportion of a particular bacterium among all bacteria studied); and, where possible, by (2) the proportion of resistant cases hospital- versus community-acquired; and (3) the frequency with which each drug is used to treat infections caused by each bacterium. (For most calculations, measure (3) is not available.) Finally, we compare and contrast each country's resistance experience and, using the subset of data comparable across the three countries, examine correlations in patterns of resistance.

These methods represent preliminary steps to gauge whether patterns of antibiotic resistance converge over time amongst countries that currently have little population interchange. Future research would benefit from better surveillance of resistance, more comparable data reporting, data on antibiotic utilization, and further methodological advances in clinically- and policy-relevant aggregation of "bug-drug" data.

## Results

### China

In 1988, the World Health Organization West Pacific Regional Office set up two antimicrobial resistance surveillance centers in Beijing and Shanghai. Meanwhile, China's Ministry of Health also established the China Nosocomial Infection Surveillance (CNIS) program, which monitors hospital-acquired infections. Unfortunately, most of the surveillance programs in China focus on urban hospitals. We lack data on urban communities and for the rural majority. Nevertheless, the available data allows us to piece together a picture of the extent of antimicrobial resistance in the most populous country in the world.

To examine AMR development in China, we use annual data from a seven-year (1994–2000) study by China's National Center for Antimicrobial Resistance, which reports resistance levels of ten most prevalent bacteria to a common antibiotic, ciprofloxacin (Table 1) [25]. With small sample sizes, the annual measured percentage of isolates found to be resistant varies considerably; to smooth the random variation attributable to small sample size, we use three-year running averages. Some bacteria such as ECO and MRSA have high proportions (60–80%) of resistant strains, whereas the prevalence of resistant strains for others such as PMI is quite low. Almost all but MSSA and PMI have shown considerable growth in resistance over the study period, resulting in an average annual growth rate of about 15%.

**Table 1:** Resistance prevalence of ten common bacteria to Ciprofloxacin in China, 1994–2000

**unit: %**											
Rank	Bacter.		1994	1995	1996	1997	1998	1999	2000	Average Resistance*	Average Growth Rate*

1	Escherichia coli (ECO)		53	49	60	61	60	63	62	***59***	***3***
2	Pseudomonas aeruginosa (PAE)		9	10	7	18	13	17	18	***13***	***17***
3	Klebsiella pneumoniae (KPN)		2	4	7	8	14	17	18	***10***	***40***
4	Staphylococci epidermidis (SEP)		22	33	34	35	41	40	46	***36***	***9***
5	Staphylococci aureus (SAU)	MRSA**	47	65	74	88	83	78	76	***76***	***7***
		MSSA**	8	18	10	5	8	20	14	***11***	***8***
6	Enterococcus faecalis (EFA)		25	34	28	34	32	45	45	***34***	***9***
7	Enterobacter cloacae (ECL)		12	9	13	14	22	31	30	***18***	***26***
8	Acinetobacter baumannii (ABA)		7	7	19	20	23	31	37	***20***	***29***
9	Citrobacter freundii (CFR)		10	21	20	17	22	26	26	***20***	***10***
10	Proteus mirabilis (PMI)		8	2	13	2	5	14	12	***7***	***10***
		***Mean***								***28***	***15***
		***Median***								***20***	***10***

* Based on three-year running averages.

** Staphylococci aureus (SAU) is further grouped as methicillin susceptible staphylococci aureus (MSSA) and methicillin resistant staphylococci aureus (MRSA).

**Figure 2:**
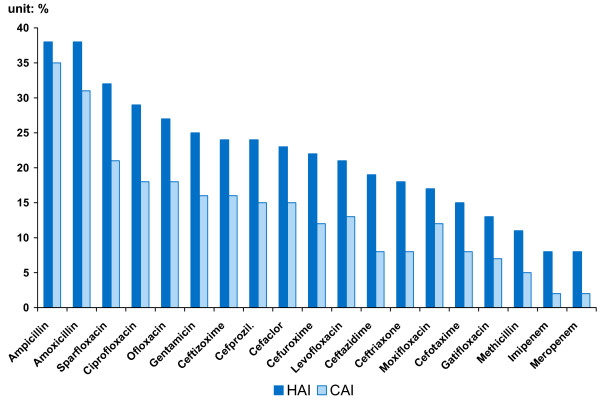
Hospital-acquired infections (HAI) are more resistant than community-acquired infections (CAI) to a wide range of antibiotics in China.

**Figure 3:**
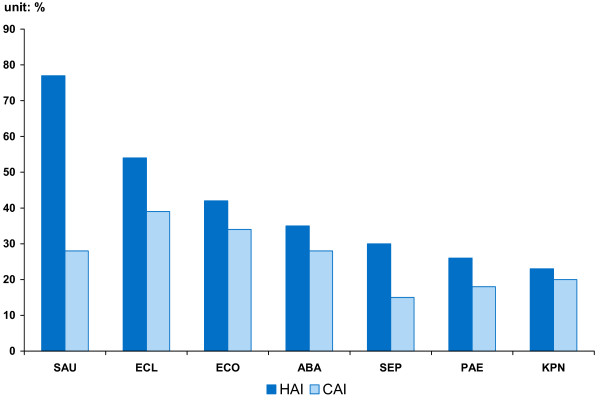
The Seven most common bacteria show higher resistance among hospital-acquired infections (HAI) than community-acquired infections (CAI) in China.

Another series of studies by the China Bacterial Resistance Surveillance Study Group focused on resistance prevalence among different patient types, i.e. those with hospital-acquired infections (HAI) versus community-acquired infections (CAI) [26,27]. We construct two measures to compare HAI and CAI resistance prevalence. First, by aggregating the seven bacteria, we get a measure γ indexed on the nineteen drugs. γ is calculated by multiplying the resistance rate of each bacterium by its isolation frequency and proportion among HAI (or CAI) infections, and then summing across bacteria. The measure is reported in the last two columns of Table 2 and graphed in Figure 2. Second, by aggregating the drugs, we obtain a measure indexed on bacteria. However, because we lack data on how often each drug is used, the best we can do is report the simple average for all drugs (implicitly assuming each drug is used with equal frequency). We name this measure Mean Resistance, shown in the last row in Table 2 and graphed in Figure 3.

**Table 2:** Resistance patterns of the seven most common bacteria for Hospital-acquired Infections (HAI) and Community-acquired Infections (CAI), China 2001

unit: %																
	
Antibiotic(s)	SAU (n = 176)		SEP (n = 84)		ECO (n = 308)		ECL (n = 78)		PAE (n = 232)		KPN (n = 215)		ABA (n = 191)		γ	
	HAI (37)	CAI (139)	HAI (14)	CAI (70)	HAI (44)	CAI (264)	HAI (27)	CAI (51)	HAI (95)	CAI (137)	HAI (48)	CAI (167)	HAI (46)	CAI (145)	***HAI***γ_*H*_	***CAI***γ_*C*_

Methicillin	89	30	43	27	n/a	n/a	n/a	n/a	n/a	n/a	n/a	n/a	n/a	n/a	***11***	***5***

Ampicillin	100	82	86	67	89	80	100	90	n/a	n/a	54	66	n/a	n/a	***38***	***35***
Amoxicillin	89	27	29	6	84	81	100	94	n/a	n/a	90	95	48	50	***38***	***31***
Ceftizoxime	87	28	14	7	32	25	96	86	n/a	n/a	33	26	96	92	***24***	***16***
Cefaclor	87	31	21	10	32	26	89	78	n/a	n/a	33	25	65	57	***23***	***15***
Cefuroxime	89	29	22	4	32	25	74	47	n/a	n/a	29	23	57	41	***22***	***12***
Cefprozil.	87	26	21	4	34	25	78	61	n/a	n/a	33	23	94	86	***24***	***15***
Ceftazidime	92	37	50	13	5	7	59	28	11	14	21	4	30	15	***19***	***8***
Cefotaxime	84	28	21	6	0	7	44	26	41	26	4	5	28	16	***15***	***8***
Ceftriaxone	89	28	21	3	9	8	48	29	40	25	6	5	33	15	***18***	***8***
Imipenem	76	21	21	1	2	0	0	2	2	3	0	1	2	1	***8***	***2***
Meropenem	78	21	14	1	2	0	0	0	2	2	0	1	2	2	***8***	***2***
Ciprofloxacin	87	35	36	30	75	53	63	33	26	13	19	14	26	17	***29***	***18***
Ofloxacin	78	30	36	30	75	55	59	31	17	15	15	14	22	17	***27***	***18***
Levofloxacin	46	7	29	10	68	52	33	20	22	15	10	11	13	12	***21***	***13***
Sparfloxacin	89	39	50	40	75	56	63	33	43	31	25	16	15	14	***32***	***21***
Moxifloxacin	5	2	14	3	64	43	22	18	43	27	4	8	13	15	***17***	***12***
Gatifloxacin	30	1	14	4	36	25	7	6	23	17	6	6	15	14	***13***	***7***
Gentamicin	87	31	36	21	43	38	30	24	37	29	27	16	35	21	***25***	***16***
***Mean Resistance***	***77***	***28***	***30***	***15***	***42***	***34***	***54***	***39***	***26***	***18***	***23***	***20***	***35***	***28***		

Both measures reinforce the finding that infections acquired in a hospital are often more drug resistant than other (community-acquired) infections. For the seven bacteria, the mean resistance rate of HAI is on average 1.5 times that of CAI in China. For the nineteen drugs, the aggregate measure of resistance for HAI, γ_*H*_, is on average 1.9 times that for CAI, γ_*C*_. This pattern is most extreme for infections caused by SAU, where resistance of HAI is two- to three- times that of CAI, depending on which measure is used. (T-tests of the difference between two groups indicate a p-value of less than 0.01 for the γ's and less than 0.09 for the mean resistance). Moreover, the prevalence of drug resistance for both kinds of infections is quite high. Mean resistance of HAI is 41% and that of CAI is 28%.

### United States

Fairly comprehensive data on resistance trends in the U.S. come from the National Nosocomial Infections Surveillance System (NNIS) for hospital-based resistance, and the U.S. Active Bacterial Core Surveillance (ABC) project, which surveys a population of 16 million to 25 million community residents in 9 states each year [28-30]. We use data from an ABC program that surveys *Streptococcus pneumoniae *(SPN) from 1997 to 2002 to examine prevalence and trends (Table 3). The average growth rate of resistance for this bacterium was 8%, lower than the 15% number for China. Interestingly, unlike the upward resistance trend in China, SPN resistance declined in the last two years of the study period in the US, following an initial rise. Such data should not be interpreted to mean that actual prevalence is permanently declining, since measurement issues engender considerable year-to-year variation in the sample prevalence.

**Table 3:** Non-susceptibilities of *Streptococcus pneumoniae *(SPN) in U.S. communities, 1997–2002

Unit: %								
Antibiotic	1997	1998	1999	2000	2001	2002	***Average Resistance***	***Average Growth Rate***

Penicillin	25	24	27	28	26	21	***25***	***2***
Cefotaxime	13	14	17	18	16	12	***15***	***-1***
Erythromycin	15	15	21	22	19	17	***18***	***4***
TMP/Sulfa	29	29	32	32	30	25	***30***	***-3***
Levofloxacin	n/a	0.2	0.2	0.3	0.7	0.5	***0.4***	***39***
Vancomycin	0	0	0	0	0	0	***18***	***8***

**Figure 4:**
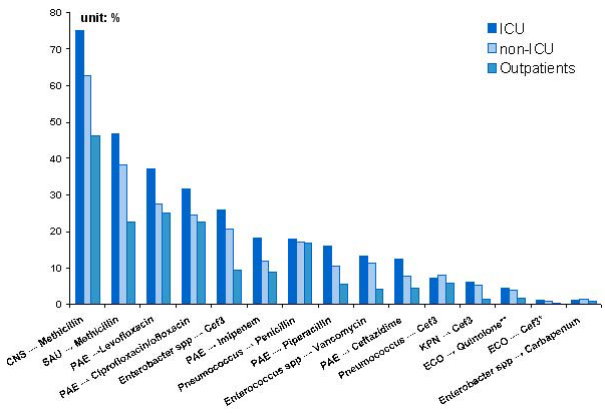
ICU patients have the highest resistance rates in selected drug-bug pairs, followed by non-ICU inpatients and outpatients, U.S. 1999–2002.

The US NNIS program provides data for inpatients and outpatients. Further, among inpatients, the NNIS differentiates between those in and not in the ICU. For almost every bug-drug pair, resistance prevalence is highest among ICU patients, followed by non-ICU inpatients, with the lowest prevalence among outpatients (Table 4 and Figure 4). This pattern seems consistent with clinical reality, since patients in ICUs are more likely to have a weak immune system, either because of prolonged treatment or their own compromised conditions; moreover, many are catheterized, offering a conduit for bacteria.

**Table 4:** Resistance prevalence for selected drug-bug pairs by patient type, U.S. 1999–2002

unit: %				
Pair	Bacterium (resistant to) → drug	ICU patients	non-ICU inpatients	Outpatients

A	PAE → Ciprofloxacin/ofloxacin	32	25	23
B	PAE → Levofloxacin	37	28	25
C	PAE → Imipenem	18	12	9
D	PAE → Ceftazidime	13	8	5
E	PAE → Piperacillin	16	11	6
F	SAU → Methicillin	47	38	23
G	Enterococcus spp → Vancomycin	13	11	4
H	ECO → Cef3*	1	1	0
I	ECO → Quinolone**	5	4	2
J	KPN → Cef3	6	5	2
K	Enterobacter spp → Cef3	26	21	10
L	Enterobacter spp → Carbapenum	1	1	1
M	CNS → Methicillin	75	63	46
N	Pneumococcus → Penicillin	18	17	17
O	Pneumococcus → Cef3	7	8	6
	***Mean***	***21***	***17***	***12***

*Cef3 (3^rd ^generation cephalosporin) = ceftazidime, cefotaxime or ceftriaxone;

**Quinolone = ciprofloxacin, ofloxacin or levofloxacin.

Compared with China, the U.S. exhibits more moderate differences in resistance prevalence among different patients. The average prevalence of resistance for ICU, other inpatients, and outpatients in the U.S. are 20%, 17% and 13%, respectively; in China, average resistance for hospital-acquired infections is 41% and that for community-acquired infections is 28%.

Pooling all patients together (Table 5), we find the prevalence of resistance and its growth to be 17% and 7% respectively, consistent with our previous observation that the U.S. seems to have both lower resistance prevalence and less dramatic increase in resistance than China does.

**Table 5:** Resistance prevalence of eight common bacteria, U.S. (all patients pooled), 1999–2002

unit: %								
Bacterium	Resistant to antibiotic(s)		1999	2000	2001	2002	***Average Resistance***	***Average Growth Rate***

PAE	Ciprofloxacin/ofloxacin		23	25	28	29	***26***	***8***
	Levofloxacin		29	30	31	30	***30***	***1***
	Imipenem		12	12	15	13	***13***	***4***
	Ceftazidime		8	8	9	9	***9***	***4***
	Piperacillin		10	10	11	12	***11***	***6***
SAU (MRSA)	Methicillin		32	35	38	39	***36***	***7***
Enterococcus spp	Vancomycin		11	8	10	10	***10***	***-1***
ECO	Cef3		1	1	1	1	***1***	***0***
	Quinolone		2	3	4	5	***4***	***36***
KPN	Cef3		4	4	4	5	***4***	***8***
Enterobacter spp	Cef3		19	19	18	19	***19***	***0***
	Carbapenum		1	1	1	1	***1***	***0***
CNS	Methicillin		60	61	62	63	***62***	***2***
Pneumococcus spp	Penicillin		14	16	19	19	***17***	***11***
	Cef3		5	8	7	7	***7***	***16***
		***Mean:***					***17***	***7***

### Kuwait

There is considerably less detailed data on antibiotic resistance for Kuwait than for China or the U.S. We gathered data on antimicrobial resistance among isolates of eight different bacterial diseases over the most recent five years. The data is based on surveillance from a single large teaching hospital, Mubarak Al-Kabeer Hospital, which serves a catchment area representing about 60% of Kuwait's population. We report that data for the first time here and in a companion paper [31] (see Tables 6, 7, 8, 9).The average resistance level for all surveyed bacteria was about 27% from 1999 to 2003 (Table 10), higher than the 17% for the U.S. and about the same as the 28% China. As for the other two countries, resistance appears to be growing in Kuwait.

**Table 6:** Resistance trend in isolates of *Salmonella *spp. over 5 years in Kuwait

Antibiotic	Percentage (%) of resistant isolates in:				
	
	1999 (n = 216)	2000 (n = 215)	2001 (n = 129)	2002 (n = 167)	2003 (n = 165)
Amikacin	0	0	0	0	0
Ampicillin	6	12	7	25	26
Amoxicillin-clavulanate	5	10	7	2	0
Cefotaxime	0	1	0	1	0
Ceftriaxone	0	1	0	2	0
Cefuroxime	1	1	0	27	41
Cephalexin	2	10	37	57	50
Chloramphenicol	8	21	0	18	18
Ciprofloxacin	0	0	14	10	16
TMP/SMX	8	8	10	20	20
Gentamicin	6	1	0	42	42
Imipenem	0	0	0	0	0
Meropenem	0	0	0	0	0
Piperacillin	6	13	13	23	25
Piperacillin/tazobactam	0	0	0	0	0

No ESBL-producing strain has been isolated so far

**Table 7:** Resistance trend in isolates of *Streptococcus pneumoniae *over a 5-year period in Kuwait

Antibiotics	Percentage (%) of resistant isolates in:				
	
	1999 (n = 78)	2000 (n = 61)	2001 (n = 73)	2002 (n = 66)	2003 (n = 90)
Cefotaxime	0	0	4	5	6
Ceftriaxone	0	0	3	5	4
Cefuroxime	0	0	8	9	41
Cephalexin	0	0	NT	NT	NT
Chloramphenicol	3	5	25	5	0
Erythromycin	16	20	23	26	30
Imipenem	0	0	0	0	0
Penicillin	32	38	46	52	54
Teicoplanin	0	0	0	0	0
Vancomycin	0	0	0	0	0

NT = not tested

**Table 8:** Percentage of *Enterococcus *species resistant to often-tested antibiotics over 5 years in Kuwait

Antibiotic	Percentage (%) of resistant isolates in:				
	
	1999 (n = 370)	2000 (n = 335)	2001 (n = 322)	2002 (n = 248)	2003 (n = 212)
Ampicillin	1	1	3	2	0
Erythromycin	59	78	77	75	92
Gentamicin	26	36	61	52	98
Nitrofurantoin	2	2	2	36	86
Norfloxacin	36	47	47	NT	NT
Penicillin	16	38	35	53	85
Teicoplanin	0	0	0	1	0
Vancomycin	1	0	0	2	0

NT = not tested

**Table 9:** Percentage of *Staphylococcus aureus *resistant to often-tested antibiotics over 5 years in Kuwait

Antibiotic	Percentage (%) of resistant isolates in:				
	
	1999 (n = 648)	2000 (n = 595)	2001 (n = 484)	2002 (n = 420)	2003 (n = 286)
Ampicillin	96	100	98	96	98
Amoxicillin-clavulanic acid	6	33	27	22	29
Cephalexin	33	30	25	36	34
Ciprofloxacin	10	35	30	45	50
Clindamycin	18	24	20	20	27
Cloxacillin	23	24	9	22	17
Erythromycin	38	34	26	28	27
Fusidic acid	NA	20	19	64	27
Gentamicin	25	21	16	24	27
Methicillin	23	24	9	22	17
Penicillin	95	95	99	96	99
Teicoplanin	0	0	0	0	0
TMP/SMX	24	27	31	18	94
Vancomycin	0	0	0	0	0

**Table 10:** Average Resistance Levels of Major Bacteria in Kuwait, 1999–2003

unit: %										
	ECO	KPN	PAE	SPN	Shigella spp.	Salmonella spp.	Enterococcus spp.	SAU	***Average Resistance***	***Average Growth***

Average Annual Resistance	13	8	5	31	45	65	37	8	***27***	***17***

## Discussion: Comparing antibiotic resistance in China, the U.S. and Kuwait

In China, resistance rates exhibit a clear and rapid upward trend. In the U.S., resistance currently appears to grow at a more leisurely pace. Kuwait seems to be somewhere in between. It is important to note that the pace of growth may depend on the whether resistance to a particular antibiotic has reached a potential equilibrium. As shown in the previous data, the 3% resistance growth rate of ECO against Ciprofloxacin in China (Table 1), is considerably lower than it is in the other two countries against similar quinolone drugs (Table 5 and Table 10). This is probably because ECO resistance may have virtually reached equilibrium in China by the beginning of the study period; hence it didn't grow much in subsequent years.

That resistance does not grow without bound highlights the importance of comparing the current prevalence of resistance in the three countries. After all, the prevalence of resistance reflects the risk of a drug-resistant infection for any given patient. A low rate of growth is small consolation if patients already face a high baseline risk of a acquiring an expensive, debilitating and even potentially untreatable "superbug" infection.

The prevalence of resistance also substantially differs across countries, although as noted previously, surveillance data is far from ideal in capturing the true scope of the problem. As shown in Table 11, using the data currently available, China has far higher prevalence of resistance for all the bacteria studied. For example, in China resistance of SPN to one of the oldest antibiotics, erythromycin, reaches 73%, while the figure for Kuwait is only 23%. A challenge for the U.S. is the exceptionally high level of Vancomycin-Resistant Enterococcus spp (VRE). In the U.S., 53% of Shigella spp are resistant to Trimethoprim/Sulfamethoxazole (TMP/SMX), in contrast to 0% in both of the other countries. These examples suggest that severity of resistance may be correlated with volume of usage. Vancomycin is less affordable in both China and Kuwait, presumably resulting in less usage in those countries.

**Table 11:** Resistance rates in China, U.S. and Kuwait, hospital surveillance data for 2001

From Tables 1,2,3,8 and 9; Unit: %					
Bacterium(a)	Antibiotic(s)	Pair	China	U.S.	Kuwait

SAU	Methicillin	A	37	38	9
SPN	Erythromycin	B	73	19	23
	Cefotaxime	C	0	16	4
*Enterococcus spp*	Vancomycin	D	4	10	0
*ECO*	Ceftazidime	E	9	1*	5
	Cefotaxime	F	18	1*	1
	Ceftriaxone	G	21	1*	1
	Ciprofloxacin/Ofloxacin	H	56	3	26
PAE	Ceftazidime	I	17	9	27
	Ciprofloxacin/Ofloxacin	J	27	28	31
KPN	Ceftazidime	K	9	4*	14
	Cefotaxime	L	17	4*	13
	Ceftriaxone	M	20	4*	13
	Ciprofloxacin	N	18	12**[27]	18
*Salmonella *spp	Amoxicillin-clavulanate	O	10	4	7
	Ceftriaxone	P	5	1	0
	Ciprofloxacin	Q	0	0.4	10
	TMP/SMX***	R	0	3	0
	Gentamicin	S	10	2	0
*Shigella *spp	Amoxicillin-clavulanate	T	35	2	20
	Ceftriaxone	U	6	0	0
	Ciprofloxacin	V	6	0	0
	TMP/SMX	W	0	53	0
	Gentamicin	X	18	0.2	0
		***Average***	***17***	***7***	***9***

* The original U.S. NNIS reported resistance rates to either one of the Cef3 drugs, i.e. ceftazidime, cefotaxime or ceftriaxone. We assume the same rates for each drug.

** Based on surveillance of ICU patients

*** TMP/SMX = Trimethoprim/Sulfamethoxazole

Table 12 compares the three countries with Japan and Taiwan regarding prevalence of three important drug-resistant bacteria: MRSA, penicillin resistant SPN (PRSP) and vancomycin-resistant *Enterococcus *spp (VRE) [32-34]. Interestingly, each country has its own most problematic resistance culprit. For China, MRSA is the biggest threat, where resistance among hospital-acquired infections reaches almost 90%, the highest among the five countries. For the U.S., VRE is high. VRE growth in the U.S. can be traced to the late 1980s and is probably among the highest in the world. For Kuwait, PRSP is considerable. Both Taiwan and Japan are also troubled by at least one of these three resistant bacteria.

**Table 12:** MRSA, PRSP & VRE in Selected Countries

Unit: %			
	MRSA (HAI only)	PRSP	VRE

China	89 (2001)	27 (2001)	0 (2001)
U.S.	16 (2001)	26 (2001)	0.3 (1989), 8 (1993), 12.8 (2001) in ICU
Kuwait	9 (2001)	46 (2001)	0 (2001)
Japan [33]	60–80% (1999)	11–40 (1999)	n/a
Taiwan [34]	n/a	69 (2000)	2 (2000)

### Resistance correlations

How similar or different are resistance patterns in different countries? Does transmission travel across national borders as humans do? If so, do countries' resistance patterns converge? To begin to examine this issue, we construct coefficients of resistance correlation among China, U.S. and Kuwait. We rank resistance rates for 24 bug-drug pairs and define perfect correlation as each bug-drug pair displaying the same resistance rank. Perfect negative correlation exists if the ranks in two countries go in precisely the opposite order. Table 13 reports the correlation coefficient for each pair of countries. The statistic by definition is bounded between -1 and 1, where -1 means perfect disagreement while 1 means perfect agreement. Thus the bigger the statistic, the more correlated two countries' resistance patterns are.

**Table 13:** Ranks of resistance rates in China, U.S. and Kuwait, 2001(Rank correlations at bottom of table)

Bacterium(a)	Antibiotic(s)	China	U.S.	Kuwait
SAU	Methicillin	*3*	*2*	*11*
SPN	Erythromycin	*1*	*4*	*4*
	Cefotaxime	*21*	*5*	*14*
*Enterococcus spp*	Vancomycin	*20*	*7*	*17*
ECO	Ceftazidime	*15*	*17*	*13*
	Cefotaxime	*8*	*18*	*15*
	Ceftriaxone	*6*	*19*	*16*
	Ciprofloxacin/Ofloxacin	*2*	*13*	*3*
PAE	Ceftazidime	*11*	*8*	*2*
	Ciprofloxacin/Ofloxacin	*5*	*3*	*1*
KPN	Ceftazidime	*16*	*9*	*7*
	Cefotaxime	*12*	*10*	*8*
	Ceftriaxone	*7*	*11*	*9*
	Ciprofloxacin	*9*	*6*	*6*
*Salmonella *spp	Amoxicillin-clavulanate	*13*	*12*	*12*
	Ceftriaxone	*19*	*20*	*18*
	Ciprofloxacin	*22*	*21*	*10*
	TMP/SMX	*23*	*14*	*19*
	Gentamicin	*14*	*15*	*20*
*Shigella *spp	Amoxicillin-clavulanate	*4*	*16*	*5*
	Ceftriaxone	*17*	*23*	*21*
	Ciprofloxacin	*18*	*24*	*22*
	TMP/SMX	*24*	*1*	*23*
	Gentamicin	*10*	*22*	*24*
***Correlation Coefficients***		***CHN_US: 0.18***	***US_KW: 0.46***	***CHN_KW: 0.60***

Of course, methods for aggregation and comparing patterns of resistance across countries and over time should be improved, and applied more fruitfully with better data from increased local and global surveillance. But even this preliminary analysis reveals some interesting patterns. For example, resistance rates in China are much more strongly correlated with those in Kuwait than those in the U.S. This correlation pattern suggests that at least in the short run, resistance in a country is more likely to be determined by endogenous factors (such as strictness of practices for prescribing drugs). In the long run, the frequency and magnitude of contacts among nations with different resistance problems is likely to be critical. Because Kuwait and China are relatively isolated countries, it is less surprising that their antibiotic resistance problems show domestic characters. However, as we expect them to be opening more to the world, particularly China, the problem may worsen when these countries can increasingly export and import antibiotic resistance. China, the most populous country in the world and an economy with the highest growth, is particularly likely to exacerbate the problem. As illustrated in Figure 1, the number of Chinese departures to overseas destinations has been growing at increasing rates in the past decade and continues to show upward momentum in recent years.

No doubt, there are also complex interactions with levels of economic well- being. Drugs become more affordable as countries become richer, but they are likely to be given out more carefully, particularly since concerns about resistance also increase. The critical question for policy is whether countries can control their own resistance problems, and also avoid importing the problem from abroad.

## Conclusion

We have outlined the nature of the antimicrobial resistance problem as an important health and cost issue for three quite disparate nations, and by inference for a broad swath of the world's population. Surprisingly, this issue virtually never receives prominent attention at the national or international level, despite its scope and potentially devastating impact on global public health in the coming decades.

We examined antimicrobial resistance data for China, Kuwait, and the United States. In each country, we looked at specific infectious agents and their resistance to particular antibiotics or other antimicrobials. Though an upward trend of resistance is found broadly, the patterns of correlation between countries' resistance rates suggest predominantly independent profiles. But we would expect greater convergence as globalization increases contacts between different nations' populations, raising questions about how to coordinate an effective international response [35].

Future research should develop better methods of data aggregation, explore the patterns of drug resistance across more countries, analyze the determinants of transmission of drug resistance across national boundaries, and assess how those determinants are progressing. Individuals everywhere would benefit if far greater attention were paid to the problem of antimicrobial resistance.

## Competing interests

The author(s) declare that they have no competing interests.

## Authors' contributions

RFZ assembled the data, carried out the analysis and drafted the manuscript. KE and RJZ conceived of the study, participated in its design and coordination, and helped to draft the manuscript. VR provided the Kuwait data and helped to draft the manuscript. All authors read and approved the manuscript.
